# Blind spots and actionable insights for urban governance of the climate–biodiversity–health nexus

**DOI:** 10.1038/s42949-026-00345-w

**Published:** 2026-02-09

**Authors:** Milutin Stojanovic, Thea Wübbelmann, Sirkku Juhola, Nadja Kabisch, Timon McPhearson, Veera Lipponen, Christopher Raymond

**Affiliations:** 1 Environmental Sciences, University of Helsinki, Helsinki, Finland; 2 Helsinki Institute for Sustainability Science (HELSUS), University of Helsinki, Helsinki, Finland; 3 Physical Geography & Landscape Ecology, Leibniz University Hannover, Hanover, Germany; 4 Urban Systems Lab, New York University, New York, NY USA; 5 Stockholm Resilience Center, Stockholm University, Stockholm, Sweden; 6 Environmental and Resource Economics, University of Helsinki, Helsinki, Finland

**Keywords:** Climate-change policy, Environmental social sciences, Sustainability

## Abstract

The critical nexus between biodiversity loss, climate change, and societal change is increasingly intertwined, requiring coordinated action, especially in urban contexts. This study examines how urban governance operationalizes the climate-biodiversity-health nexus in four European case cities through a goals-oriented framework informed by the Planetary Health approach. We conduct a qualitative analysis of urban policy documents to assess the degree of change and level of coordination across climate mitigation, adaptation, biodiversity, and health domains. While cities have employed transformative solutions like nature-based solutions (NBS), we consistently identify policy blind spots such as fragmented mid-level targets, sectoral silos, insufficient attention to indirect emissions, and reliance on soft governance tools. We conclude by offering actionable insights for transformative urban nexus governance: mainstream transformative metrics and indicators, create new institutional innovations, integrate multi-benefit NBS across sectors, expand governance toolkits to address trade-offs, and co-create a culture of innovation, learning, co-creation and leadership.

## Introduction

In the face of looming climate change and biodiversity crises, cities are increasingly recognized as key actors for climate action^1^. Cities have played a central role in urban climate governance including designing the institutions, infrastructures, and behaviors necessary for climate adaptation and carbon mitigation^2–4^. However, policy analyses over the past decade have identified limitations in the comprehensiveness and level of integration of adaptation and mitigation in existing climate action plans^5^. Scholars are thus increasingly investigating the climate and health nexus^6,7^, as well as the climate and biodiversity nexus in cities^8,9^, with increasing recognition that urban governance needs to consider the critical interconnections, feedbacks, and dynamic relationships between biodiversity, climate change, and health. The recent joint report of the Intergovernmental Panel on Climate Change (IPCC) and Intergovernmental Science-Policy Platform on Biodiversity and Ecosystem Services (IPBES) calls out the interconnections between climate and biodiversity crises and their interdependence with other societal issues, including health^10^.

Feedback loops and interactions among biodiversity, climate and society at multiple spatial, temporal, and organizational scales can transmit risks from one system to another and thus need to be considered together as a nexus^11^. The feedback between biodiversity and other elements in the nexus can be negative, positive and contain both positive and negative influences^12^. Governance strategies that ignore Climate-Biodiversity-Health (CBH) nexus interactions may significantly underestimate the transmitted risks from interconnected global challenges, and also miss out on opportunities for transformative climate change mitigation and adaptation, as well as producing measurable biodiversity and wellbeing co-benefits^13,14^.

While recent studies highlight the opportunities and challenges of transformative governance within the CBH nexus^11^, empirical investigations into how these interactions within the nexus manifest in real-world governance contexts remain limited and largely anecdotal. There is a notable lack of systematic empirical investigations into what is happening in urban governance of the CBH nexus, and in a more general sense, what governance mechanisms are capable of effectively anticipating and addressing the interlinked challenges of climate, biodiversity, and societal change. A recent nexus assessment of EU policy documents reveals that most policy interlinkages are climate-centered, i.e., they consist of the expected contribution of circular economy and biodiversity policies to climate mitigation/adaptation^15^. Further, there is a dearth of policy analyses that have reported on city-level strategies and actions for addressing co-benefits and co-determinants across the CBH nexus^16,17^.

The aim of this paper is to empirically explore how the urban governance of the CBH nexus is approached in policy and planning practice. The aim is guided by the following objectives: (1) Apply a structured policy document analysis to investigate how policy strategies, plans and targets across European case cities envision and engage with the CBH nexus; (2) Critically examine what type of actions cities propose to address climate change, biodiversity loss, and public health and well-being needs, and how transformative these actions are; and (3) Identify bright spots and blind spots resulting from employing a nexus approach in urban governance to enable distillation of key insights forward for improving governance for the CBH nexus.

We use qualitative policy analysis to assess the degree of transformative change in urban environmental governance and identify insights and paths forward across the CBH nexus. We used a stratified sample representing the four largest biogeographical regions of Europe—Atlantic, Continental, Mediterranean, and Boreal to identify case studies to examine BCS nexus governance in practice. We examine four urban areas—Cork, Klagenfurt, Päijät-Häme, Malta—within the EU, which provide sample cases with similar overarching regulatory frameworks important to empirical comparison. Though focused on EU cases, results have potential global significance due to EU influence and power through markets to affect governance approaches far outside the region. We examine policies and plans that span two or more domains of the nexus and explore the most synergistic and transformative examples of nexus governance to identify bright spots (and blind spots) for learning and applying in other urban contexts globally.

**Fig. 1:**
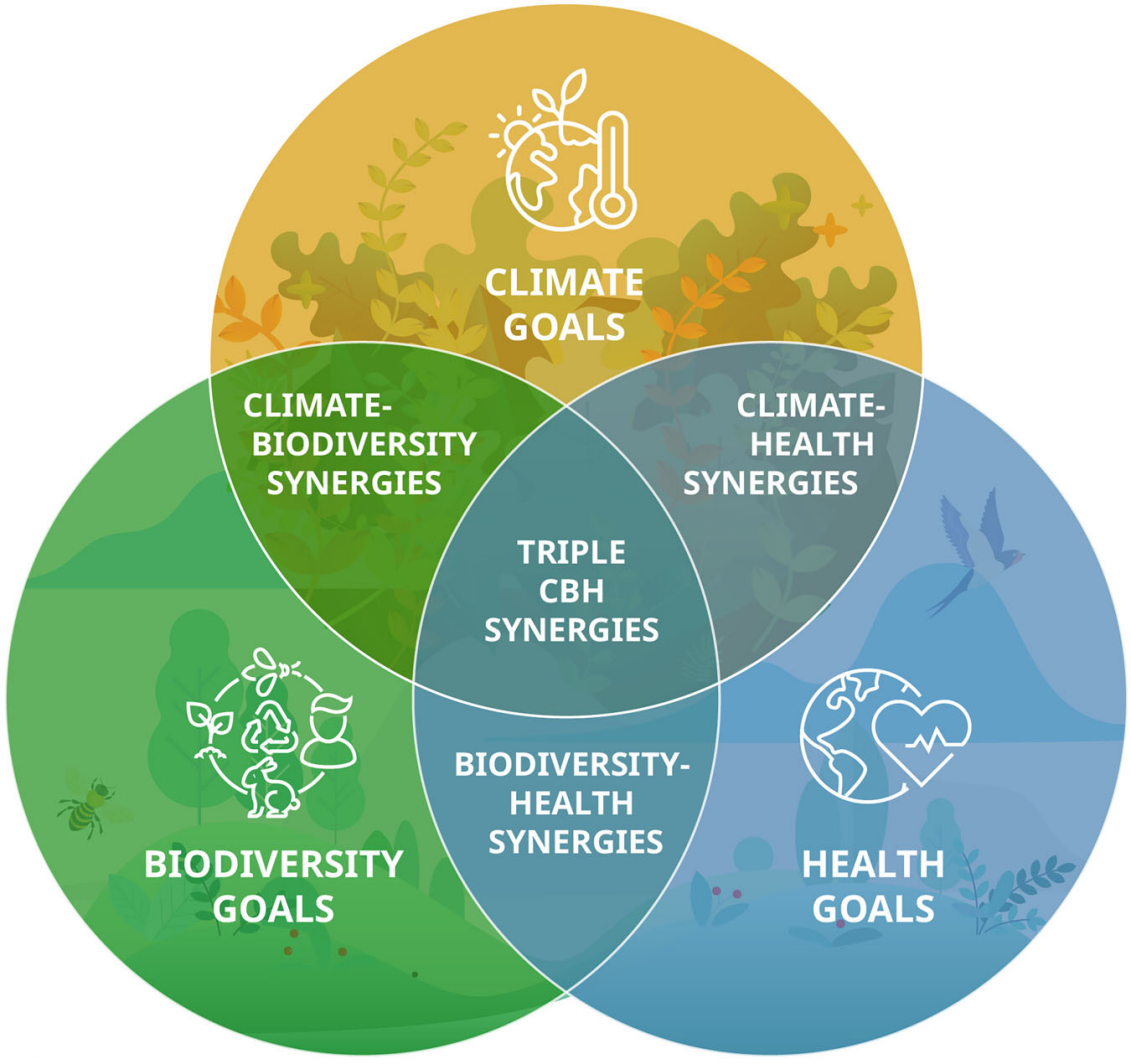
The climate-biodiversity-health nexus approach to governance.

Our analytical framework for the CBH Nexus (Fig. 1) builds on recent conceptualizations of the CBH nexus, which focus on identifying the key domains and illustrating how they interact with each other^13^. It was proposed by ref. ^18^ to set clear sustainability objectives, but here we build on its use by ref. ^19^ to support the delivery of integrated planning and policy objectives.

The health dimension focuses on the interplay between human health and well-being and associated justice and governance challenges^11^. Important direct and indirect effects exist across biodiversity, climate and society, denoted as “couplings”. The biodiversity-climate coupling refers to strategies that provide co-benefits or costs for climate and biodiversity objectives; for example, the use of nature-based solutions in cities to mitigate the risks of coastal flooding and to enhance biodiversity outcomes^20^. Much research has also focused on the carbon sequestration potential of different types of ecosystems, implying a co-benefit between biodiversity and carbon mitigation^21–23^. Yet, some carbon mitigation strategies can be detrimental to biodiversity^24,25^, implying that the biodiversity-climate nexus can have both positive and negative direct and indirect effects.

The climate-health coupling denotes how climate action produces co-benefits and costs for mental and physiological health. For example, health benefits are received by avoiding the psychological stress of natural disasters^26^, and the health benefits derived from reduced air pollution^27^ or heat stress^28^. Yet certain climate actions can adversely affect human health; for example, the planting of certain tree species can lead to the local production of allergens^29,30^ and impact mental health^31^.

The biodiversity-health coupling has been captured through multiple frameworks and concepts. For example, the One Health framework ensures that human, animal (e.g., domesticated, livestock), and environmental health (including wildlife health) questions are evaluated holistically^32,33^. The ecosystem services framework shows how ecosystems contribute to human wellbeing through provisioning, regulating, supporting, and cultural services^34^. Additionally, literature on green equity and environmental justice seeks to spotlight the inequitable access to and exposure to the benefits of quality green space, which directly and indirectly affect health outcomes in society^35^. Marsell et al.^36^ provide a framework for connecting both the beneficial as well as harmful links between biodiversity and health. Reducing harms is connected to the provision of medicines and decreasing air and noise pollution exposures; restoring capacities such as attention restoration and stress reduction; building capacities such as promoting physical activity; and reducing the causes of harm, such as reducing exposure to dangerous wildlife, zoonotic diseases and allergens.

As an all-encompassing framework, here we consider the CBH nexus through the theoretical lens of the Planetary Health framework to examine the interconnections between human health, ecosystem integrity, and climate stability in urban governance. Grounded in the definition of planetary health as “the health of human civilization and the state of the natural systems on which it depends”^37^, the framework provides a means for understanding how urban environments contribute to or mitigate environmental degradation and health inequities^38^. By applying a nested model of Planetary Health tailored for urban settings^39,40^ this study emphasizes the interdependence of human well-being, biodiversity, and climate resilience in cities. It builds on calls for ambitious, integrated, and city-level sustainability transformations to safeguard both human and planetary health^41^, offering a theoretical grounding to explore how urban governance can be directed toward more holistic and sustainable outcomes across the CBH nexus.

Despite the paramount importance of sustainability transformations, most definitions do not specify how these processes can be observed empirically or measured, and with what methods^42^. Since a goals-oriented framework could be more easily operationalized and applied^18^, we develop an analytical framework for analyzing urban transformations based on defining four overarching goals of urban sustainability governance: climate mitigation, climate adaptation, biodiversity protection, and human health and well-being. We then deconstruct each of these four overarching CBH goals into a set of action fields cities can use to achieve each of them (see Table 1 for the full list of strategies). The action fields are identified based on the operational question “What types of actions are available to cities to achieve each of the overarching goals?”, grounded in the respective literature on climate, biodiversity and health governance^40,43,44^, and empirically adjusted through a preliminary policy analysis. We consider this operational focus crucial as it enables our goals-oriented framework to instantiate the concept of sustainability transformations in specific, observable and measurable policy actions, which can then be evaluated for their impact on the progress toward each of the four overarching CBH goals. By operationalizing a “nexus approach to governance”^13^ in our goals-oriented framework^18^, this methodological approach enables empirical investigation of city transformations in the CBH nexus and mapping how nexus approach to governance looks like in cities.

**Table 1:** List of action fields available to cities to achieve the CBH goals

Climate Mitigation	Climate Adaptation	Biodiversity	Health & well-being
M1. Clean energy production	A1. flood protection	B1. nature protection	H1. Providing access to nature
M2. Efficient buildings	A2. temperature moderation	B2. nature restoration and conservation	H2. enabling active lifestyles
M3. carbon sinks	A3. food system resilience	B3. urban greenery	H3. reducing noise, air and light pollution
M4. waste management	A4. organizational adaptation	B4. preventing invasive alien species	H4. supporting sustainable diets
M5. sustainable transport	A5. climate change education	B5. reducing chemical pollution	H5.institutionalizing the planetary health approach
M6. private sector decarbonization		B6. raising awareness on biodiversity	
M7. promoting climate action			

We study the degree of change in urban governance of mitigation, adaptation, biodiversity, and health and well-being in the case cities. To assess the transformative potential of policy plans, targets and strategies, the method used here builds on the sustainability transformations literature, particularly in urban contexts. Determining the degree of change is challenging, particularly in identifying a transformation, as transformative changes are often recognized only in hindsight^45^. We build on the analytical framework for assessing the degree of change in climate governance^46^, expand the definition to cover the whole CBH nexus, and adapt it to our goals-oriented framework by including the potential of policies to satisfy the CBH needs. We draw on three degrees of change across the CBH nexus (incremental, reformistic, and transformative change), which cover climate, biodiversity and health policies of cities. Incremental change refers to small-scale, technocratic improvements made within existing policy frameworks and institutional logics, often lacking systemic impact. Reformistic change refers to more ambitious, systemic adjustments that seek to realign institutional practices or policy priorities while still operating within the current governance paradigm. Transformative change refers to deep, structural shifts in governance, societal values, and institutional arrangements that not only redefine sustainability pathways but also demonstrate a capacity to effectively address environmental and social challenges. (For detailed elaboration on these distinctions, see section “Methods”). We use these definitions to empirically examine what kind of changes are advocated in the actual urban governance of the nexus.

## Results

**Fig. 2:**
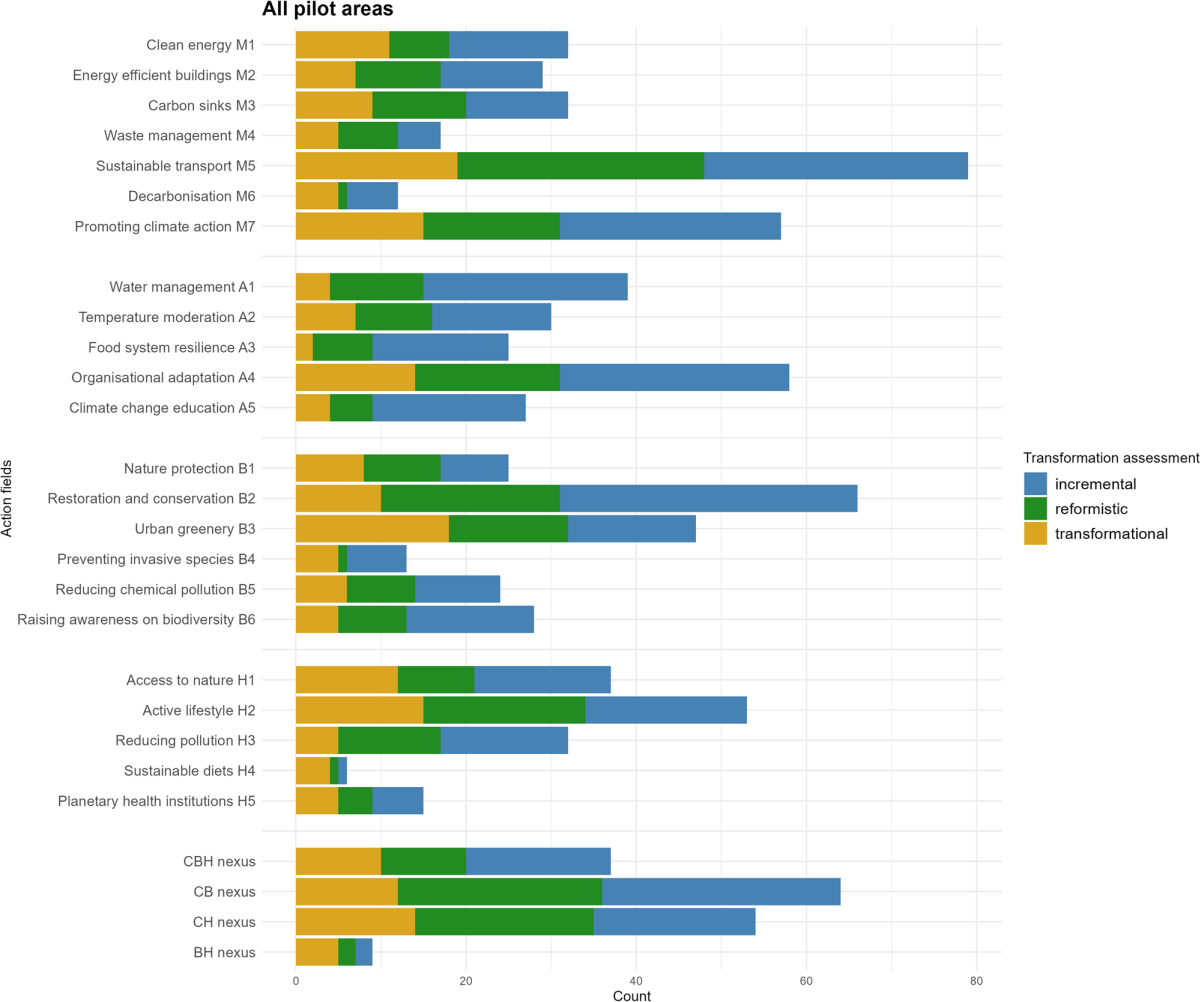
Total number of entries for each action field combined across case cities, including the degree of change.

Based on the analysis of 32 strategic planning documents, we coded 362 policy plans in the four case areas, with the highest number of plans concerning mitigation (258 plans), then biodiversity (203), adaptation (179), and health (143), with 127 plans covering two domains (i.e., having double synergies: climate-biodiversity (64); climate-health (54), biodiversity-health (9)), and 37 plans having triple CBH synergies, with 59 plans including nature-based solutions (Fig. 2). We identified 23 action fields which are available to cities to address the overarching CBH goals. On average, we found ca. 30 entries per action field (see Fig. 4). Most common action fields include: sustainable transport (M5: 79 entries), promoting climate action (M7: 57), organizational adaptation (A4: 58), nature restoration (B2: 66), urban greenery (B3: 47), and promoting active lifestyles (H2: 53 entries). Strategies that were identified with a lower number of entries are waste management (M4: 17), private sector decarbonization (M6: 12), controlling invasive alien species (B4: 13), and supporting sustainable diets (H4: 6).

**Fig. 3:**
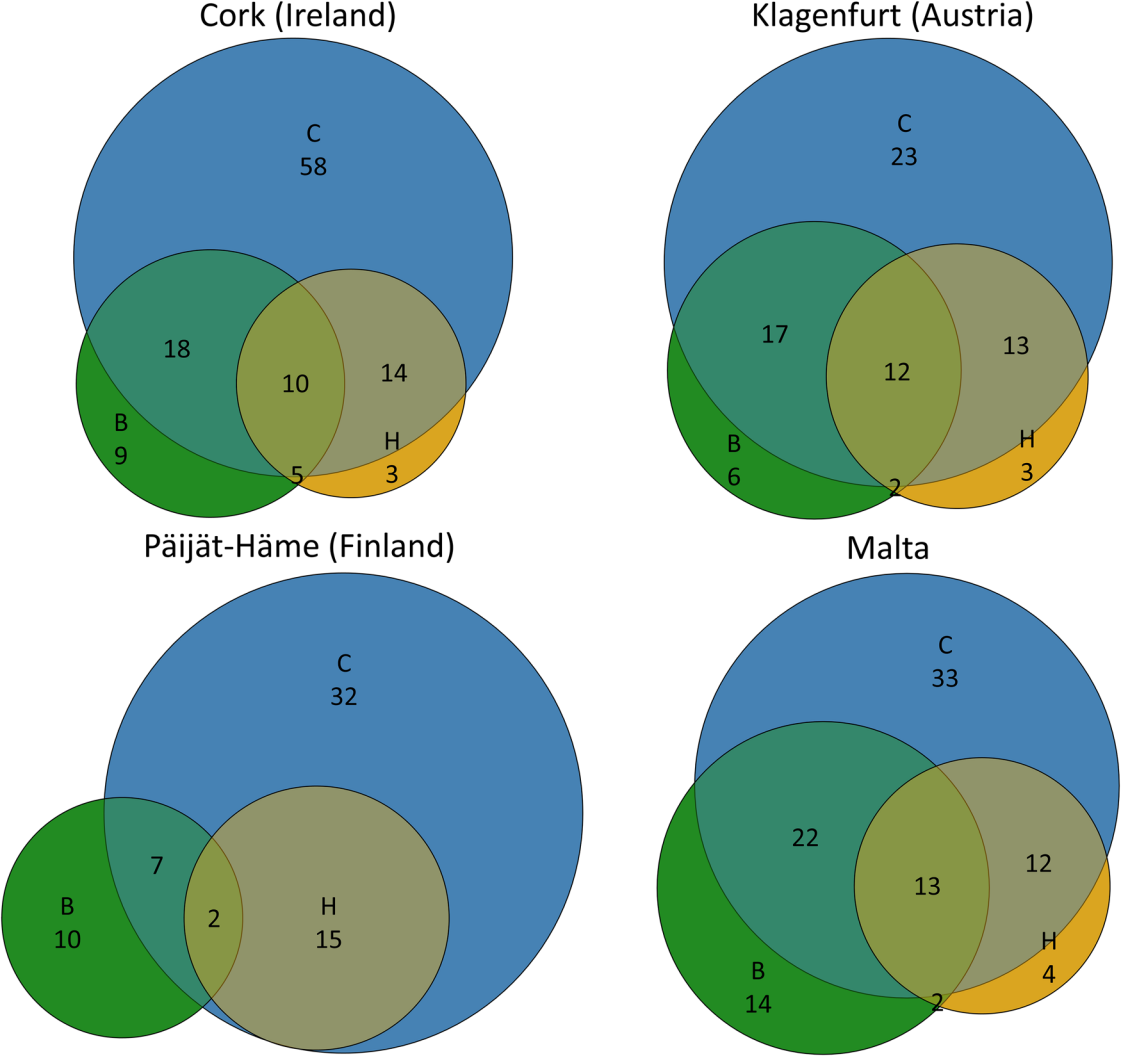
Number of entries falling in each nexus domain. The size of circles and intersections represent the number of entries for each nexus domain in the four case cities. C climate, B biodiversity, H health.

**Fig. 4:**
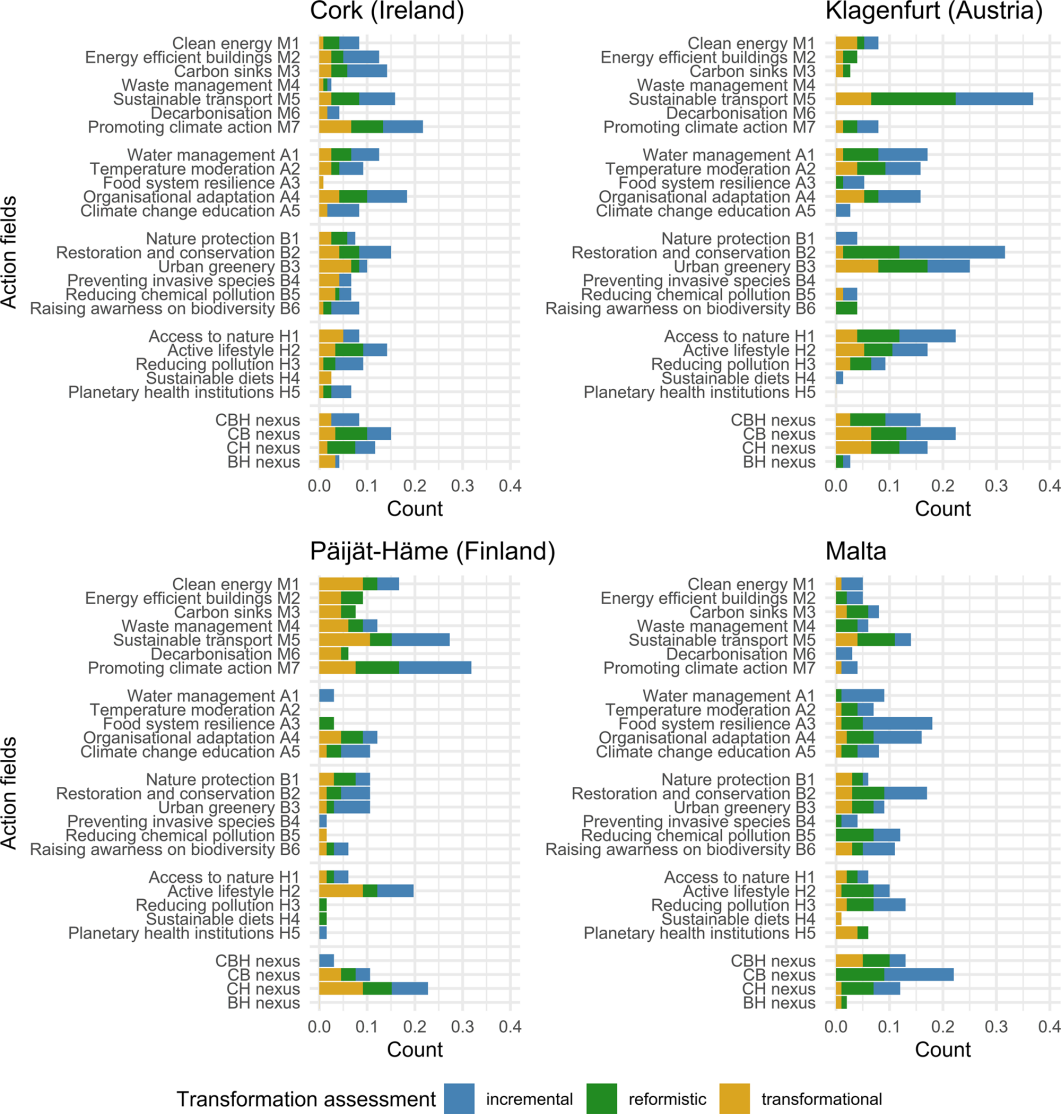
**A-4.D**. Percentage share of entries within each action field per case city, including the degree of change.

All the analyzed cities contain vocabularies and objectives representing and targeting the CBH nexus. In general, the existing plans primarily promote incremental and reformistic change such as promoting climate action and increasing green infrastructure, but also suggest many transformational proposals such as radical modal shifts toward sustainable mobility. Figure 3 highlights how each city prioritizes different domains of the CBH nexus, revealing variation in thematic focus and degrees of integration. Figure 4 complements this by illustrating differences in the number and type of action fields used to address the CBH nexus, and shows that not all areas of the CBH nexus are equally covered by different cities. Specifically, every city focuses on a particular area of the CBH nexus, typically two out of the four overarching goals, where their governance has transformative characteristics. Cork excels in climate adaptation and health and well-being; Klagenfurt in climate mitigation and biodiversity protection; Päijät-Häme shows signs of transformative governance in mitigation and biodiversity; Malta in biodiversity and climate adaptation. Importantly, most overarching goals show transformative potential (e.g., climate neutrality by 2030; or stopping biodiversity loss), meaning that they are nominally on track to achieve the UN targets. All analyzed urban areas emphasize policies with double and triple synergies across the three domains of the CBH nexus. Overall, cities highlight synergistic plans, notably green infrastructure that contributes to mitigation, adaptation, biodiversity and health and well-being, as well as other no regret solutions such as improving energy efficiency of buildings or reducing traffic pollution.

While our four urban case areas demonstrate efforts toward transformative change in CBH governance, we identified several blind spots, limiting the effectiveness and transformative potential of how a CBH nexus approach to urban governance is practiced. We define “blind spots” as systemic gaps or limitations in urban governance where the integration of climate, biodiversity, and health goals is absent, insufficient, or poorly operationalized, thereby constraining the capacity of CBH nexus approaches to deliver transformative outcomes. These blind spots highlight challenges in policy implementation, sectoral integration, trade-offs management, and policy-impact strategies.

### Limited policy coherence and implementation gaps

While all cities have developed CBH nexus-oriented policy goals, there is often a disconnect between high-level sustainability ambitions and mid-level implementation strategies. Key issues include notably a lack of specificity in targets— many policy documents outline broad ambitions (e.g., carbon neutrality by 2030, expanding urban greenery) but fail to provide detailed and realistic plans on how to achieve and implement them. The main blind spot concerns fragmented or inconsistent mid-level goals as some cities set conflicting or weak intermediate targets, insufficient for achieving the proclaimed overarching goal. For example, Malta plans for electrifying only 50% of vehicle transport without reforming clean energy production, and Cork plans for deep energy-efficiency renovation of only 50% of residential buildings, while at the same time aiming for the carbon neutrality target.

A related issue is unclear funding and resource allocation—in most cases, policies lack detailed financial plans, making it difficult to assess their feasibility and implementation capacity. All cities emphasize ambitious CBH nexus targets, notably for mitigation, but are in the need of developing strategies to bridge high-level goals with implementation measures.

### Persistence of sectoral silos and weak institutional integration

Despite efforts to establish cross-sectoral governance structures, many cities seem to continue to operate in departmental silos, limiting the effectiveness of the nexus approach. A key issue that appeared through the policy analysis is the incompatibility among some policy plans written by different government departments (e.g., urban expansion projects that conflict with biodiversity goals) which points to a potential insufficient institutional integration. Despite the establishment of new governance bodies (e.g., task forces, advisory councils), these bodies often function alongside traditional departments rather than replacing outdated separated structures.

### Insufficient recognition of indirect emissions and systemic trade-offs

Most cities focus on direct emission reduction goals and localized sustainability interventions, neglecting the broader systemic impacts of urban consumption patterns. A key gap concerns the failure to address indirect emissions, as policies often overlook emissions embedded in food systems, industrial production, and imported goods, despite their major climate and biodiversity footprints.

There is a narrow focus on green infrastructure in all case areas. While green infrastructure is widely promoted as the prime example of nexus governance, cities rarely address broader land use trade-offs (related to e.g., urban sprawl, population increase, agricultural expansion, or deforestation linked to bioenergy production). The narrow focus on green infrastructure creates two problems. First, in pursuing ambitious mitigation targets, there is a pressure for externalizing emissions by accounting them outside the city. In Lahti (Päijät-Häme), biomass burning is promoted as a “renewable” clean energy source and it is the stepping stone for carbon neutrality. However, wood burning actually increases carbon emissions compared to coal in the first 100 years if the wood is replanted sustainably^47^, but despite this, because wood emissions are accounted for where the wood was cut, this is considered a mitigation “victory” for Lahti. The second issue is that these patterns unravel potential and undisclosed contradictions or misalignment among sustainability goals. Some policies pursue carbon neutrality without considering biodiversity trade-offs, such as large-scale tree planting initiatives that may reduce local species diversity when species diversity is not considered in tree species selection^48^.

### Over-reliance on voluntary and soft governance measures

Cities frequently rely on informal strategies, persuasion, awareness campaigns, and voluntary citizen initiatives rather than binding formal regulations or structural changes of political and economic systems^49^. The lack of formal regulation can result in weak to non-existent enforcement mechanisms leaving many sustainability strategies to rely on awareness-building and creating moral incentives, rather than enforceable regulations. In rare cases redesigning economic incentives is employed.

Associated with soft governance measures, a lack of accountability and monitoring is a pervasive issue in the policy documents and city administrations. There is often insufficient tracking of policy effectiveness, making it difficult to assess progress toward CBH goals. The reliance on soft governance measures discloses critical dependence on behavioral change rather than systemic reform. Many policies encourage voluntary action (e.g., promoting individual or corporate climate actions such as sustainable diets or low-carbon economy) rather than mandating shifts (e.g., regulating high-emission food supply chains). Some key policies depend on strategies for change lacking robust empirical or theoretical backing. For example, Cork plans to have 50% of all trips done by active or public transport, yet there are no regulatory or economic measures sanctioning or even significantly incentivizing against the use of cars. Consequently, many policy plans have weak effectiveness, insufficient for transformative change^50,51^.

### Scarcity of types of nexus solutions

Despite the high-level emphasis in IPCC-IPBES reports of the crucial role of nexus policies^10,11,13^, we found few types of synergistic solutions in the studied case areas, consistent with the lack of synergistic solutions reported in recent literature. Except for a few types of NBS and solutions for sustainable transport-active mobility, both of which have rather limited potential for climate and biodiversity goals, there is a remarkable lack of synergistic governance solutions articulated for cities. Particularly lacking are solutions that explicitly target climate mitigation while also advancing biodiversity conservation or public health outcomes. This absence points to a critical implementation gap: while the discourse around synergies is well established, the design and institutionalization of cross-cutting policies remain underdeveloped. The result is a continued reliance on single-goal policies combined with a handful of nexus solutions, that fall short of the transformative ambition required by the calls for “a nexus approach to governance”^10^.

## Discussion

Across the four study cities, several governance-related characteristics appear to enable more transformative forms of action within the CBH nexus. These include the existence of cross-sectoral coordination structures that align environmental, health, and planning departments (e.g., Cork’s Climate Action Coordination Group; Päijät-Häme’s regional partnerships); stable political leadership and institutional continuity, which support long-term visioning and experimentation (as evident in Klagenfurt’s climate strategy development); and evidence-based planning and stakeholder co-production, which enhance legitimacy and social learning (notably in Malta’s green infrastructure planning). Collectively, these governance arrangements foster reflexive, integrated, and adaptive decision-making—key attributes identified in the literature as prerequisites for transformative governance^10,11,13^.

While these governance enablers help explain why the study cities are able to implement more transformative forms of CBH action, it is equally important to recognize that not all urban contexts possess comparable institutional capacities or political momentum. In many cases, transformation unfolds gradually through smaller, cumulative adjustments that strengthen cross-sectoral coordination, policy coherence, and citizen engagement. While our analysis focuses on transformative change, the study also reveals that incremental and reformistic actions often provide essential entry points for advancing CBH nexus governance and create the enabling conditions for deeper systemic change. Incremental improvements—such as the integration of climate and health indicators into monitoring systems, modest coordination among sectoral departments, or awareness-raising campaigns—can generate early co-benefits and institutional learning. Reformistic actions, including cross-sectoral policy alignment, participatory planning mechanisms, and the establishment of funding streams for nature-based interventions, further institutionalize these early gains and expand organizational capacity. As ref. ^11^ emphasize, incremental changes alone are unlikely to be scaled up without broader institutional shifts, yet they can still pave the way for transformation by fostering collaboration, data sharing, and political buy-in.

While our analysis emphasizes the need for transformative governance, many of the study cities also demonstrate how incremental and reformistic actions can serve as practical entry points for advancing CBH nexus integration. Incremental actions include the development of cross-sectoral planning strategies that explicitly connect mobility, climate, health, and biodiversity objectives; sustained interdepartmental negotiations to identify meaningful compromises and align budgetary cycles (Patterson et al., 2021); and the strategic investment in agents capable of linking multiple policy domains and action moments across climate, biodiversity, health and mobility policies. Cities can also enhance policy relevance by ensuring that urban plans reflect the lifestyle needs of diverse resident profiles, including sufficiency-oriented lifestyles (Dunkel et al., 2025). Reformistic changes, by contrast, focus on reshaping institutional rules and value systems. These include addressing constitutional and legal constraints to cross-sectoral governance—such as the balance between property rights, human rights, and the right to a healthy environment (Patterson et al., 2021; Soininen et al., 2025)—and rethinking which values are prioritized in strategic planning, encouraging public institutions to account for a fuller suite of nature’s values, particularly relational, intrinsic, and shared ones (Kenter et al., 2025). By embedding such incremental and reformist measures into policy practice, cities can gradually strengthen their institutional capacity and cultural readiness for the more ambitious transformative shifts required to address the interconnected challenges of climate change, biodiversity loss, and public health.

The extent to which cities can advance transformative CBH governance is strongly mediated by their position within multilevel governance systems and the legal competences assigned to them. While all study cities operate under EU regulatory frameworks, their authority to act independently varies. For instance, Lahti benefits from significant municipal discretion in environmental management and has used this autonomy to pilot biodiversity–health initiatives such as “health forests” and “biodiverse daycares.” Malta, by contrast, operates within a more centralized national structure but leverages strong alignment with EU cohesion and environmental funding programs to implement large-scale nature-based and biodiversity projects. Cork and Klagenfurt exhibit more constrained legal mandates for cross-sectoral interventions but compensate through participatory governance, regional partnerships, and the use of evidence-based planning.

Cities clearly possess substantial capabilities to design and implement integrated CBH solutions; however, the rule of law can also pose substantial obstacles to achieving environmental and health-related goals (Soininen et al., 2025). Whether law is supportive or restrictive of climate, biodiversity, and health outcomes depends heavily on the context in which it is applied—its institutional interpretation, enforcement capacity, and interaction with other policy domains. The pursuit of CBH policies is thus influenced by endogenous factors, such as the presence of change agents and leaders able to bridge governance silos, the availability of funding, and the strength of political will, as well as by exogenous factors, including the constraints and opportunities created by EU law, trade treaties, and broader geopolitical conditions. Together, these legal, institutional, and contextual differences help explain why some cities have advanced further toward transformative CBH governance, while others remain limited to incremental or reformist change. They also underscore the importance of institutional flexibility and multilevel coherence in enabling cities to align local innovation with broader regulatory and policy frameworks.

This paper identified a number of blind spots concerning the governance of the BCS nexus across the four cases. In the remainder of this discussion, we focus on actionable insights for increasing the transformative potential of urban governance across the CBH nexus, summarized in Table 2. These responses synthesize empirical insights from the four case cities and supported by the literature, respond directly to the limitations and blind spots identified in the current governance practices. Overall, these insights provide an actionable roadmap for urban actors seeking to operationalize the CBH nexus through context-sensitive pathways that link incremental and reformistic initiatives to transformative governance.

**Table 2:** Blind spots in urban governance of the BCS nexus and the actionable insights to overcome them

Summary of blind spots	Actionable insights to overcome the blindspots
1. Limited policy coherence and implementation gaps	1. Mainstream transformative metrics and indicators
2. Persistence of sectoral silos and weak institutional integration	2. Institutionalize cross-sectoral nexus governance
3. Insufficient recognition of indirect emissions and systemic trade-offs	3. Expand governance Toolkits and evaluation to address systemic trade-offs
4. Over-reliance on voluntary and soft governance measures	4. Cultivate a culture of innovation, learning, co-creation and leadership
5. Scarcity of types of nexus solutions	5. Integrate multi-benefit nature-based solutions across sectors

### Mainstream transformative metrics and indicators

Cities need to move beyond broad sustainability commitments—like carbon neutrality by 2030 or biodiversity net gain—and develop quantified, time-bound, and actionable mid-level targets. Cities should also develop intermediate, sector-specific targets that link high-level ambitions with operational measures^20,52^. In our sample, although certain links could be observed between high-level biodiversity conservation ambitions and individual urban greening measures, there was a systemic lack of clear, quantified mid-level targets and actionable implementation pathways for both biodiversity and climate mitigation goals—undermining the ability of cities to translate strategic ambitions into measurable outcomes. Clear implementation pathways, supported by adequate financing and institutional commitment, are essential for translating ambition into impact. Transformation cannot be monitored or managed without clear, measurable, and integrated indicators that capture synergies and trade-offs across the CBH domains. Cities could thus adopt metrics that go beyond carbon accounting to include biodiversity outcomes (e.g., native species cover and ecological connectivity) and social well-being (e.g., access to green space, respiratory health, and mortality rates). These indicators could be institutionalized in planning and budgeting processes to support accountability and strategic decision-making.

### Institutionalize cross-sectoral nexus governance

Urban governance has traditionally operated through fragmented departmental structures, where climate, health, transport, and biodiversity policies are designed and implemented in isolation, what undermines efforts to deliver co-benefits, manage trade-offs, and align strategies with systemic sustainability goals^53,54^. Institutional innovation and bridging organizations—including the establishment of cross-departmental task forces, decision institutions, policy arenas and interdepartmental collective actions—enables more collaboration around sustainability initiatives, as well as foster coordination, shared accountability, and mutual learning^55–57^. For example, Cork’s formation of interdepartmental working groups and coordination units for climate action improved alignment between adaptation, mobility, and green infrastructure strategies. Similarly, Päijät-Häme’s regional collaboration structures have supported the development of climate roadmaps that integrate biodiversity and health concerns across municipal boundaries.

Research shows that such integrative governance arrangements are vital for addressing complex, “wicked” problems like those situated at the climate–biodiversity–health nexus. The literature on polycentric governance highlights the importance of overlapping and interacting institutions to manage multi-scalar, cross-sector challenges effectively^58^. Urban climate governance gains effectiveness when embedded in flexible, multilevel institutional arrangements that enable experimentation, cross-sector collaboration, and civil society engagement^4^. Moreover, ref. ^3^ emphasize the role of institutional capacity for collaborative governance, which includes not only formal structures but also shared visions, routines of cooperation, and the ability to negotiate across competing objectives. They call for urban administrations to foster cross-sector collaboration and policy integration in turbulent environments that facilitate coherence among sectoral strategies and better reflect the interdependencies across sustainability domains. In sum, institutional innovation is not merely about structural reorganization, but about cultivating governance cultures that value integration, co-benefits, and long-term resilience. By reforming governance architecture and promoting integrative routines, cities could enhance their transformative capacity to act across the CBH nexus.

### Expand governance toolkits and evaluation to address systemic trade-offs

Urban CBH strategies tend to focus on direct emissions or local biodiversity—such as place-based interventions, transport modal shifts or energy retrofits—while often neglecting systemic impacts like resource flows and global consumption, thereby neglecting indirect emissions and biodiversity loss from energy, material and food imports, and global land use change. Cities should go beyond isolated actions, such as green infrastructure and voluntary behavior change, by developing new, evidence-based governance tools that explicitly manage trade-offs—especially in land use, energy, industry, and food systems. Incorporating indirect emissions and planetary boundaries into urban planning is critical. Expanding the evidence base will also require investments in monitoring and evaluation, to go beyond direct emissions sources to better understand and evaluate indirect emissions and impacts across systems and their interactions.

To increase their transformative potential, cities must expand their governance toolkits to better account for and address indirect emissions—by regulating and incentivizing low-carbon, biodiverse, and health-promoting urban metabolisms across supply chains and consumption systems. This may include food policy, procurement standards, and embedded emissions assessments designed to reduce carbon leakage and address emissions embodied in imported goods and services^59^. Embracing systems-thinking in policy design is crucial for cities to move from incremental improvements to structural and transformational shifts in how urban life interacts with planetary boundaries^54,60^.

### Cultivate a culture of innovation, learning, co-creation and leadership

Nexus governance requires a commitment to multiple modes of innovation, learning, and knowledge co-creation across administrative sectors. Drawing on the results of a systematic review of 250 nature-based solutions projects, ref. ^61^ identify good practice criteria for the delivery of integrated solutions, including: (i) Effective—the ability to deliver intended benefits and address specific challenges; (ii) Inclusive—the incorporation of diverse perspectives, stakeholders, and communities in planning; (iii) innovative—capacity to transform challenges into opportunities for creative and novel approaches; (iv) locally appropriate—tailored to local conditions and integrating site-specific knowledge and resources; (v) multifunctional—provide diverse benefits simultaneously; (vi) sustainable in the long-term— provide diverse benefits simultaneously over the long-term, and (vii) up-scalable—involving experimenting and adapting designs based on local contexts. Our CBH nexus assessment reveals the critical importance of new forms of leadership for addressing the interest conflicts, power games, legitimacy concerns and trade-offs between equally desirable goals, including integrative leadership to ensure policy integration across the nexus^62^. For example, in Cork, innovative leadership initiatives—such as the mayor and government officials commuting by bicycle, retrofitting government buildings to net-zero standards, transitioning the municipal vehicle fleet to electric, and eliminating single-use plastics at government events—demonstrate a proactive, integrative approach to embedding sustainability principles and values into everyday governance practice.

### Integrate multi-benefit nature-based solutions across sectors

Nature-based solutions (NBS) such as urban greenery, wetlands, urban forests, green roofs, and permeable pavements should be strategically designed to deliver simultaneous benefits for climate mitigation, adaptation, biodiversity, and health^63^. Embedding multifunctional green infrastructure into city planning as high-impact, low-regret strategies enhances systemic resilience and maximizes co-benefits across the CBH nexus^64^. Expanding green-blue infrastructure not only mitigates urban heat island effects and flood risk, but also enhances ecosystem services, air quality, and mental well-being. Empirical evidence suggests that multifunctional NBS are among the most frequently used and impactful nexus interventions in urban planning^65,66^. However, cities could move beyond ad hoc greening and develop systemic, spatially integrated green infrastructure plans that prioritize ecological connectivity and social equity^67^. The five actionable insights presented in this study—mainstreaming transformative metrics, institutional innovation, expanding governance toolkits, fostering a culture of co-creation and leadership, and integrating multi-benefit nature-based solutions—collectively offer a roadmap for unlocking the transformative potential of urban governance across the CBH nexus. By directly addressing persistent blind spots, they highlight how cities can move beyond incremental adjustments toward more systemic, synergistic, and measurable sustainability transitions. These insights not only synthesize empirical patterns across diverse urban contexts but also emphasize how targeted interventions, institutional reforms, and holistic planning can align planetary and human health goals.

Nevertheless, several limitations must be acknowledged. First, many impactful urban governance solutions—such as reducing global consumption or restructuring economic incentives—fall outside the operational reach of CBH synergies. Second, cities often lack the institutional capacity, financial resources, and jurisdictional authority needed to implement integrated and transformative strategies at scale. These limitations underscore the need for future research in four critical areas: (1) governance mechanisms that enable cities to influence system-wide drivers of unsustainability, (2) multi-scalar institutional designs that strengthen local capacities for nexus actions, (3) innovative tools for monitoring indirect impacts and trade-offs, and (4) empirical studies that ground-truth text-based analyses through interviews and co-production with city practitioners. Addressing these gaps is essential for realizing the full potential of urban areas as engines of transformative change across the CBH nexus.

## Methods

### Goal specification

We break down the overarching goal of mitigation into seven action fields, representing mid-level goals available to the city government to achieve the overarching goal. The city strategies are developed based on reviewing the relevant literature, siphoned through our goals-oriented framework, and guided by the operational question “what types of actions are under jurisdiction of urban government and available to cities to achieve the overarching goal?” We follow the same procedure for adaptation, biodiversity, and health goals (see Table 1 for the list of all strategies in the framework). For every city, each policy target or plan is classified to one or more of these strategies, which identifies the aimed goal(s) and gives us its position in the CBH nexus. Each entry is then evaluated for how transformative it is, using the analytical framework for the degree of change described above. The action fields then enable us to assess city progress toward the four overarching planetary health goals, focusing on the processes under the power of a city to influence. By operationalizing a “nexus approach to governance”^13^ in our goals-oriented framework and complementing it with transformation assessment, this methodological approach enables comprehensive assessment of city transformations in relation to mitigation, adaptation, biodiversity and health goals, as well as how the city is positioned in the nexus.

### Case selection

Due to practical limitations related with the availability of city strategies and the relatively large number of documents per each city needed for analysis of the CBH nexus, we selected four urban case cities : Cork City (Ireland), Klagenfurt (Austria), Päijät-Häme region (Finland), and city-state Malta. Our case areas represent a stratified sample of the four biggest European biogeographical regions spanning the continent on East-West and North-South axis: from Atlantic to Continental, and from Mediterranean to Boreal. All our case areas are in the EU in part due to the advantages of a shared legislative framework facilitating comparative analysis, but importantly because the EU, via the so-called “regulatory imperialism”, has the potential to externalize regulatory power through markets, thereby making other cities, states and corporations conform to the EU’s regulatory standards^68^, and additionally because the EU has the most developed legislative framework in the world for environmental governance^69^, including both climate, biodiversity and public health. Our third selection criteria was a developed CBH policy discourse. We selected these case cities due to their frontrunner status, to discover what a nexus approach to governance looks like in practice in some of the most promising examples. Cork, Klagenfurt, and Lahti (the biggest city of Päijät-häme region) are all signatories of the Climate Neutral and Smart Cities contract, committing them to carbon neutrality by 2030. Malta and Päijat-häme are extensively committed to Natura 2000 cities and biodiversity protection. Cork and Lahti are champions of public health and had recently participated in a Horizon Europe project focused on the nexus between urban biodiversity and health and well-being. Additionally, it appears that small and wealthy cities are more effective at implementing novel climate and biodiversity policies^70^.

### Content analysis

**Fig. 5:**
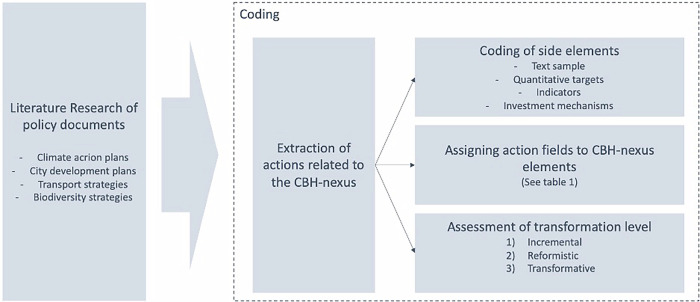
Steps undertaken for the policy analysis.

We followed a content analysis approach^71^ involving the five steps of organizing the data, sorting the data, understanding the data, interpretation and explanation of the data (Fig. 5). The material we analyzed consists of climate, biodiversity, transport, pollution control, city development, and health and well-being strategies and plans of the selected urban areas. We collected the documents concluding February 2025. In cases where there were multiple versions of the same plan (e.g. biodiversity strategy) we included only the latest version. We had 7 to 14 documents per city (Supplementary Material, Table 1). Document length varied from 10 pages to 750 pages, with an average length of approximately ca. 100 pages. The utilized material reflects both what the cities have already implemented and plan to do in the near future. The strategic documents create the framework in which climate, biodiversity, and public health work is done in the cities, but some of these are also political documents that advertise the image of the city, implying that the actual actions may be more or less ambitious than the policy plans.

We coded policy plans and targets in the CBH nexus using the Microsoft Excel program. The material was coded separately for each city. The plans and targets were coded in four main categories representing the four overarching policy goals: mitigation, adaptation, biodiversity, and health and well-being. Each of these overarching goals are broken down into five to seven possible strategies to achieve a given goal: seven urban policy strategies to achieve climate mitigation, five strategies to achieve adaptation goals, six for biodiversity, and five urban governance strategies for health and well-being goals (see Table 1 for the full list). Every entry was coded to one or more of these mid-level policy goals (e.g., a plan for urban greenery (B3) including both temperature moderation (A2) and providing access to nature (H1)), spanning one or more of the overarching goals (in this example: biodiversity, climate adaptation, and health and well-being). Policy plans with double or triple synergies across the CBH nexus (e.g., climate-health or biodiversity-health) were coded in CB, CH, BC, and CBH categories. Supplementary information coded includes: the source document introducing the policy, a text sample, specific quantitative targets of the policy (if available), proposed indicators (if available), and investment mechanisms (if available).

Every entry is coded for the degree of change (from incremental, over reformistic, to transformative) according to the definitions below. Coding qualitative data involves a significant degree of judgment, even more so when evaluating progress toward multiple and potentially diverging environmental goals, making many entries not straightforward to code, especially those that were potentially transformational. We resolved these challenges by using the EU directives as benchmarks for evaluating whether the cities’ goals and targets are transformative enough (for evaluation criteria across domains and the related EU & WHO directives see Supplementary Material, Table 2). Furthermore, if an action contained at least some transformational objectives, it was coded as transformational.

### Defining incremental, reformistic, and transformative strategies

Typical actions coded as incremental include various adjustments to maintain business as usual, with minor potential to achieve the given goal or without changing the structures and socio-economic patterns that contribute to the given problem^72^. In the context of climate mitigation goals, this can mean improving energy efficiency of buildings or investing in renewables on a scale which is not significantly different from the normal pace of economic growth, including the efficiency improvements and energy changes it brings. Early warning signals or ordinary flood defenses are also in this category. When it comes to biodiversity, incremental change can be an increase in urban greenery through sporadic green roofs and walls, or voluntary campaigns for reducing environmental pollution. In terms of health and well-being, incremental change can be minor increases of access to nature or of walking and cycling lanes. Also, the existence of nominally ambitious targets (e.g., carbon neutrality) without developed plans for how to actually get there, is also coded as incremental (i.e., it improves urban governance only as much as it expresses the ambition, without satisfying other conditions required to make impactful changes). Awareness-raising campaigns in all four domains relying on voluntary changes in the behavior of citizens of businesses are classified as incremental.

We coded as reformistic change policies that make the current system more flexible, but retain the fundamental structures or functions of the system^73^. These are the plans that potentially can assist the desired goal, but need to be further expanded and complemented. For example, if the combustion fuel to produce energy is changed or if the increase in green or recreational spaces does not outpace the population growth. Regulatory changes which mandate provision of green spaces in new developments or moderate industrial pollution are typically also in this category. Given our goals-oriented framework, in evaluating reformistic as well as other degrees of change, a lot depended not just on the type but also on the scale at which a policy is planned.

Finally, transformation is broadly considered to be a fundamental change where the patterns, elements, and interrelations of the system change more comprehensively^42^. These processes of structural changes often happen through accumulation of actions^72^, including fundamental alterations in sense-making world views, political and power relations, social networks and ecosystems, physical infrastructure and technology. It can also involve rethinking the division between urban and nature, how the urban economy works, fundamental change in citizen lifestyles or how the city is governed. Following our goals-oriented approach, we complement the standard definition of transformation by including actions that facilitate achieving the relevant goal in the CBH nexus— making the definition of transformation ultimately practical. To an extent, evaluating transformation was possible only partially on the level of individual entries, and a more comprehensive assessment was possible only by systematically evaluating the progress of a city toward the overarching goals.

There are three points to note about our analytical framework. First, according to our nested model of transformative change, the climate, biodiversity and health domains are interconnected with each other and individual policies often span goals across multiple domains, with different co-benefits, complicating the transformation assessment. In these cases, the assessment reflects the effectiveness of the policy to achieve its main goal. Transformation does not have to happen equally across all domains, but is sufficient that the change is fundamental for the main aim of the policy. Second, identifying change empirically is difficult^42^ and a strict classification into categories such as those above can be arbitrary. Hence, we consider the degrees of change more as a continuum of change, rather than a strict classification scheme. To complement these subtleties, we consider our qualitative analysis critical. Finally, we concentrate on the change advocated by the city government, while various degrees of change are possibly advocated, and are taking place outside of the formal policy sector, which is not captured in our sample.

## Supplementary information

Supplementary tables

## Data Availability

The data that supports the findings of this study are available at https://osf.io/7gxzh/files/rqtyd.
