# Supplementary material for: Blind spots and actionable insights for urban governance of the climate–biodiversity–health nexus
Source: NPJ Urban Sustain. 2026 Feb 9;6(1):42. doi: 10.1038/s42949-026-00345-w (PMC12995720; doi:10.1038/s42949-026-00345-w)
Supplement: Supplementary file 1 — Supplementary tables [file 42949_2026_345_MOESM1_ESM.pdf]

Table 1. City Documents Analyzed in the Nexus Database

City / Region	Document Title	Institution / Author	Link
**Cork**	Climate Action Charter 2019	Cork City Council (2019)	https://publications.corkcity.ie/view/768004668/
**Cork**	Climate Change Adaptation Strategy 2019-2024	Cork City Council (2019)	https://www.corkcoco.ie/sites/default/files/2021-11/cork-county-council-climate-adaptation-strategy-2019-2024-pdf.pdf
**Cork**	Cork 2050: realizing the full potential	Cork County Council and Cork City Council (2017)	https://www.corkcoco.ie/sites/default/files/2022-03/cork-2050-executive-summary-pdf.pdf
**Cork**	Cork City Climate Action Plan 2024–2029	Cork City Council (2023)	https://publications.corkcity.ie/view/90275165/
**Cork**	Cork City Development Plan (2022–2028)	Cork City Council (2022)	https://publications.corkcity.ie/view/935101702/
**Cork**	Cork Metropolitan Area Transport Strategy 2040	Cork City Council (2020)	https://www.nationaltransport.ie/wp-content/uploads/2021/06/Cork-Metropolitan-Area-Transport-Strategy-CMATS-2040.pdf
**Cork**	Heritage and Biodiversity Plan 2021-2026	Cork City Council (2020)	https://www.corkcity.ie/media/yezpuryv/heritage-biodiversity-plan-2021-2026.pdf
**Klagenfurt**	Mobilitätskonzept Klagenfurt 2035 mit Schwerpunkt ÖPNV	Traffix Verkehrsplanung GmbH (2019)	https://www.klagenfurt.at/stadtservice/mobilitaet-parken/mobilitaetskonzept-klagenfurt-2035
**Klagenfurt**	Leitbild Klagenfurt am Wörthersee 2022	Magistrat der Landeshauptstadt Klagenfurt am Wörthersee (2022)	https://www.klagenfurt.at/fileadmin/user_upload/Stadt_Klagenfurt/03-StadtINFOS/Leitbild/Leitbild_2022_Klein.pdf

**Klagenfurt**	Smart City Climate Strategy Klagenfurt am Wörthersee	Magistrat der Landeshauptstadt Klagenfurt am Wörthersee (2023)	https://www.klagenfurt.at/fileadmin/user_upload/Stadt_Klagenfurt/01-StadtSERVICE/Klima-Umwelt/Smart_City_Strategie/Smart_City_Klimastrategie_Version_7.1.pdf
**Klagenfurt**	Smart City Climate Strategy Klagenfurt on Wörthersee - Anhang	Magistrat der Landeshauptstadt Klagenfurt am Wörthersee (2023)	https://www.klagenfurt.at/fileadmin/user_upload/Stadt_Klagenfurt/01-StadtSERVICE/Klima-Umwelt/Smart_City_Strategie/Anhang_Smart_City_Klimastrategie_Version_7.1.pdf
**Klagenfurt**	4. Monitoringbericht: Smart City Klimastrategie 7.0	Magistrat der Landeshauptstadt Klagenfurt am Wörthersee (2023)	https://www.klagenfurt.at/fileadmin/user_upload/Stadt_Klagenfurt/01-StadtSERVICE/Klima-Umwelt/Smart_City_Strategie/5.Monitoringbericht_Smart_City_Klimastrategie.pdf
**Klagenfurt**	Stadtentwicklungskonzept Klagenfurt - Stadtentwicklungsstrategie 2035	Magistrat der Landeshauptstadt Klagenfurt am Wörthersee (2023)	https://www.klagenfurt.at/fileadmin/user_upload/Stadt_Klagenfurt/02-StadtVERWALTUNG/Stadtentwicklung/Stadtentwicklungskonzept_2025_/STEK_Strategie_2023_11_22.pdf
**Klagenfurt**	Masterplan Radfahren Klagenfurt	Amt der Kärtner Landesregierung & Magistrat der Landeshauptstadt Klagenfurt (2021)	https://www.klagenfurt.at/stadtservice/mobilitaet-parken/mobilitaetskonzept-klagenfurt-2035
**Klagenfurt**	Climate City Contract 2030 Climate Neutrality Action Plan	Klagenfurt & Net Zero Cities	https://netzerocities.app/resource-4062
**Päijät-Häme**	Päijät-Hämeen vihreän siirtymän ohjelma	Päijät-Häme county	https://paijat-hame.fi/wp-content/uploads/2024/04/Vihrean_siirtyman_ohjelma_web29042024.pdf
**Päijät-Häme**	Carbon Neutral Päijät-Häme 2030: Climate Action Roadmap	Päijät-Häme county	https://paijat-hame.fi/wp-content/uploads/2022/03/Climate_Action_Roadmap_english-update.pdf
**Päijät-Häme**	Lahden ilmasto-	City of Lahti	https://www.lahti.fi/uploads/2023/06/f6892f41-lahden-ilmasto-ohjelma-2023.pdf

	ohjelma 2023-2030		
**Päijät-Häme**	Nature Step to Health: Lahti Regional Health and Environment Programme 2022–2032	Päijät-Häme county	https://paijatha.fi/wp-content/uploads/2024/11/luontoaskel_terveyteen-ohjelma_2022-2032_asiakirja.pdf
**Päijät-Häme**	Jatkuvasti päivitettävä kaupunkiseutusuunnitelman ja rakennemallin toimenpidetaulukko	Päijät-Häme county	https://paijat-hame.fi/wp-content/uploads/2023/05/20230524_Jatkuvasti_paivitettava_KSS.pdf
**Päijät-Häme**	Lahden kestävän energian ja ilmastonmuutoksen toimenpidesuunnitelma vuoteen 2030	City of Lahti	https://www.lahti.fi/tiedostot/lahden-kestavan-energian-ja-ilmastonmuutoksen-toimenpidesuunnitelma-2030-secap/
**Päijät-Häme**	Kestävän kaupunkiliikkumisen ohjelma 2021-2025	City of Lahti	https://www.lahti.fi/tiedostot/kestavan-kaupunkiliikkumisen-ohjelma-sump-2021-2025-pdf/
**Malta**	A National Health Systems Strategy for Malta 2023 – 2030	Ministry for Health and Active Ageing (2022)	https://health.gov.mt/wp-content/uploads/2023/04/A_National_Health_Systems_Strategy_for_Malta_2023_-_2030_Investing_Successfully_for_a_Healthy_Future_EN.pdf
**Malta**	Action Plan Addressing Escape From Confinement of Invasive Alien Species (IAS) of Union Concern	Ministry for the Environment, Climate Change and Planning (2022)	https://era.org.mt/wp-content/uploads/2020/06/Action-Plan-Addressing-Escape-from-Confinement-of-IAS-of-Union-Concern.pdf

**Malta**	Conservation objectives and measures for Malta's marine Natura 2000 sites	Environment and Resource Authority (2023)	https://era.org.mt/wp-content/uploads/2023/02/MPAs-Conservation-Objectives-and-Measures_final_Feb2023.pdf
**Malta**	Green Paper on Greening Buildings in Malta	Ministry for the Environment, Climate Change and Planning (2020)	https://era.org.mt/green-paper-on-greening-buildings-in-malta-initiatives-for-green-walls-and-roofs-for-residential-commercial-and-industrial-buildings/
**Malta**	Malta's Sustainable Development Strategy for 2050	Ministry for the Environment, Energy, and Regeneration of the Grand Harbor (2024)	https://sustainabledevelopment.gov.mt/wp-content/uploads/2024/06/Maltas-Sustainable-Development-Strategy-for-2050-1.pdf
**Malta**	A National Health Systems Strategy for Malta 2023 – 2030	Ministry for Health and Active Ageing (2022)	https://health.gov.mt/wp-content/uploads/2023/04/A_National_Health_Systems_Strategy_for_Malta_2023_-_2030_Investing_Successfully_for_a_Healthy_Future_EN.pdf
**Malta**	Action Plan Addressing Escape From Confinement of Invasive Alien Species (IAS) of Union Concern	Ministry for the Environment, Climate Change and Planning (2022)	https://era.org.mt/wp-content/uploads/2020/06/Action-Plan-Addressing-Escape-from-Confinement-of-IAS-of-Union-Concern.pdf
**Malta**	Conservation objectives and measures for Malta's marine Natura 2000 sites	Environment and Resource Authority (2023)	https://era.org.mt/wp-content/uploads/2023/02/MPAs-Conservation-Objectives-and-Measures_final_Feb2023.pdf
**Malta**	Green Paper on Greening Buildings in Malta	Ministry for the Environment, Climate Change and Planning (2020)	https://era.org.mt/green-paper-on-greening-buildings-in-malta-initiatives-for-green-walls-and-roofs-for-residential-commercial-and-industrial-buildings/

**Malta**	Malta's Sustainable Development Strategy for 2050	Ministry for the Environment, Energy, and Regeneration of the Grand Harbor (2024)	https://sustainabledevelopment.gov.mt/wp-content/uploads/2024/06/Maltas-Sustainable-Development-Strategy-for-2050-1.pdf
**Malta**	National Climate Change Adaptation Strategy	Ministry for Resources and Rural Affairs (2012)	https://climate-laws.org/documents/national-climate-change-adaptation-strategy_e9fb?id=national-climate-change-adaptation-strategy_5bf12
**Malta**	National Air Pollution Control Program	Environment and Resources Authority (2020)	https://era.org.mt/wp-content/uploads/2021/04/NAPCP.pdf
**Malta**	National Biodiversity Strategy and Action Plan to 2030 (draft)	Environment and Resources Authority (2023)	https://era.org.mt/wp-content/uploads/2023/02/NBSAP-public-consultation-draft-19.01.23.pdf
**Malta**	National Strategy For Preventing and Mitigating the Impact of Invasive Alien Species (IAS) in the Maltese Islands	Environment and Resources Authority & Ministry for the Environment, Climate Change and Planning (2020)	https://era.org.mt/wp-content/uploads/2020/06/National-Strategy-for-IAS-Measures-for-Implementation.pdf
**Malta**	National Strategy for the Environment 2050	Ministry for the Environment, Energy and Enterprise (2022)	https://era.org.mt/nse2050/
**Malta**	National Transport Master Plan 2025	Transport Malta (2016)	https://transportmalta.wetransfer.com/downloads/c0ed2f41cbdb8b47a22ef7c932bbebaf20170327084338/cfb89a
**Malta**	National Transport Strategy 2050	Transport Malta (2016)	https://transportmalta.wetransfer.com/downloads/02a871c2a36e859b751f40dd405a4b4a20161202212452/4df52a
**Malta**	Noise Action Plan Malta	Environment and Resources Authority (2023)	https://era.org.mt/wp-content/uploads/2023/12/Noise-Action-Plan-Agglomeration-Interactive.pdf

	Agglomeration (2019 – 2024)

Table 2. Evaluation Criteria for City Transformations Assessment

Overarching Goal	Evaluation Criteria	Benchmarks / Reference Frameworks
**1. Mitigation**	Assessment of the city's overarching mitigation goal and specific plans across each mitigation strategy (M1–M7). High performance in one area can compensate for lower progress in others.	- 55% GHG reduction by 2030 vs. 1990 levels (EU Green Deal 2019; European Climate Law 2021)<br>- Targets for decarbonization across key areas: energy production (M1), buildings (M2), transport (M3), etc.
**2. Adaptation**	Assessment of city plans based on attention to key local vulnerabilities, resilience of societal and governance structures, and use of NBS to mitigate natural disasters (flood protection, temperature moderation).	- EU Climate Adaptation Strategy (2021)<br>- Nature Restoration Regulation (2024)
**3. Biodiversity**	Extent and quality of nature restoration and ecosystem protection in city plans.	- EU Biodiversity Strategy (2020): reverse ecosystem degradation by 2030<br>- Nature Restoration Regulation (2024): binding targets for ecosystem restoration<br>- EU Zero Pollution Action Plan (2021): reduce pollution to levels not harmful to ecosystems

**4. Health and Well-being**	Assessment adapted to local context, based on:<br>- Accessibility to green spaces<br>- Provision of recreational space<br>- Reductions in pollution harmful to health.	- WHO Urban Green Spaces (2017): access to green spaces ≥0.5–1 ha within 300m of homes<br>- WHO GAPPA (2018): global action plan for physical activity<br>- EU Zero Pollution Action Plan (2021): reduce air, water, soil, noise pollution to levels not harmful to human health by 2030 and 2050 vision

Table 3. Key Policy References Table

Policy / Strategy	Document Info	Link
**EU Green Deal (2019)**	Communication From The Commission: The European Green Deal. European Commission. Document 52019DC0640.	https://eur-lex.europa.eu/legal-content/EN/TXT/?uri=CELEX%3A52019DC0640

**European Climate Law (2021)**	Regulation (EU) 2021/1119 of the European Parliament and of the Council of 30 June 2021 establishing the framework for achieving climate neutrality and amending Regulations (EC) No 401/2009 and (EU) 2018/1999 ('European Climate Law'). Official Journal of the European Union L 243/1.	https://eur-lex.europa.eu/legal-content/EN/TXT/?uri=CELEX:32021R1119
**EU Climate Adaptation Strategy (2021)**	Forging a climate-resilient Europe - the new EU Strategy on Adaptation to Climate Change. European Commission. Document 52021DC0082.	https://eur-lex.europa.eu/legal-content/EN/TXT/?uri=COM:2021:82:FIN
**EU Biodiversity Strategy (2020)**	EU Biodiversity Strategy for 2030. European Commission. Document 52020DC0380.	https://eur-lex.europa.eu/legal-content/EN/TXT/?uri=celex%3A52020DC0380

**Nature Restoratio n Law (2024)**	Regulation (EU) 2024/1991 of the European Parliament and of the Council of 24 June 2024 on nature restoration and amending Regulation (EU) 2022/869 (Text with EEA relevance). Official Journal of the European Union. Document 32024R1991.	http://data.europa.eu/eli/reg/2024/1991/oj
**WHO Urban Green Spaces (2017)**	Urban green spaces: a brief for action. World Health Organization. Regional Office for Europe. ISBN: 9789289052498.	https://iris.who.int/bitstream/handle/10665/344116/9789289052498-eng.pdf?sequence=1
**WHO Urban Green Spaces (2017a)**	Urban green space interventions and health: A review of impacts and effectiveness. Full report. World Health Organization.	https://cdn.who.int/media/docs/librariesprovider2/euro-health-topics/environment/urban-green-space-intervention.pdf?sfvrsn=a2e135f3_1&download=true
**WHO GAPPA (2018)**	Global action plan on physical activity 2018–2030: more active people for a healthier world. World Health Organization. ISBN: 9789241514187.	https://iris.who.int/bitstream/handle/10665/272722/9789241514187-eng.pdf?sequence=1

**EU Zero Pollution Action Plan (2021)**	Towards a Zero Pollution for Air, Water and Soil (and annexes). European Commission. Document 52021DC0400.	https://eur-lex.europa.eu/legal-content/EN/TXT/?uri=CELEX%3A52021DC0400&qid=1623311742827
